# Curcumin attenuates renal interstitial fibrosis of obstructive nephropathy by suppressing epithelial-mesenchymal transition through inhibition of the TLR4/NF-кB and PI3K/AKT signalling pathways

**DOI:** 10.1080/13880209.2020.1809462

**Published:** 2020-08-31

**Authors:** Zhaohui Wang, Zhi Chen, Bingsheng Li, Bo Zhang, Yongchao Du, Yuhang Liu, Yao He, Xiang Chen

**Affiliations:** Department of Urology, Xiangya Hospital, Central South University, Changsha, PR China

**Keywords:** Inflammation, unilateral ureteral obstruction, lipopolysaccharides, transforming growth factor beta 1

## Abstract

**Context:**

Renal interstitial fibrosis (RIF) is characterized by the accumulation of inflammatory cytokines and epithelial-mesenchymal transition (EMT). Curcumin exerts antifibrogenic, anti-inflammatory and antiproliferative effects.

**Objective:**

To explore the mechanisms underlying the effects of curcumin on RIF.

**Materials and methods:**

Eight-week-old male C57BL/6 mice were intragastrically administered curcumin (50 mg/kg/day) for 14 days after undergoing unilateral ureteral obstruction (UUO) operations. Renal function (blood urea nitrogen [BUN] and serum creatinine [Scr]) and inflammatory cytokine levels were tested using colorimetric assays and ELISA, respectively. EMT markers were evaluated through immunohistochemistry, western blotting and qPCR. Transforming growth factor beta 1 (TGF-β1; 10 ng/mL) and lipopolysaccharides (LPS; 100 ng/mL) were used to stimulate EMT and an inflammatory response in human renal proximal tubular epithelial (HK-2) cells, respectively, for further investigation.

**Results:**

*In vivo*, curcumin significantly improved the levels of BUN and Scr by 28.7% and 21.3%, respectively. Moreover, curcumin reduced the levels of IL-6, IL-1β and TNF-α by 22.5%, 30.3% and 26.7%, respectively, and suppressed vimentin expression in UUO mice. *In vitro*, curcumin reduced the expression of vimentin and α-smooth muscle actin in TGF-β1-induced HK-2 cells. In LPS-induced HK-2 cells, curcumin decreased the release of IL-6, IL-1β and TNF-α by 43.4%, 38.1% and 28.3%, respectively. In addition, curcumin reduced the expression of TLR4, p-PI3K, p-AKT, p-NF- κB and p-IκBα in both LPS- and TGF-β1-induced HK-2 cells.

**Discussion and conclusions:**

Curcumin repressed EMT and the inflammatory response by inhibiting the TLR4/NF-κB and PI3K/AKT pathways, demonstrating its potential utility in RIF treatment.

## Introduction

Obstructive nephropathy and obstructive uropathy are two frequently-used terms to describe a renal disease that induces hydronephrosis due to the anatomy or an injury, which can occur throughout the urinary tract from the renal tubules to the urethral meatus (Klahr 2000; Bascands and Schanstra 2005). A result of an obstructive uropathy is a renal interstitial fibrosis (RIF), which features several major pathophysiologic changes, including an inflammatory response, oxidative stress, cytokine release (including interleukin (IL)-6, IL-1β and tumour-necrosis factor (TNF)-α), an epithelial-mesenchymal transition (EMT), and deposition of fibroblasts and the extracellular matrix (ECM), which leads to fibrogenesis (Wang et al. 2014; Chuang et al. 2015). EMT is the key mechanism involved in RIF progression, and is an important biological process whereby epithelial cells lose their cell adhesion and cell polarity characteristics and develop several mesenchymal characteristics, such as migration and invasion (Kalluri and Weinberg 2009; Liao and Yang 2017).

The inflammatory response is an important risk factor that can predict progression towards end-stage renal failure (Pulskens et al. 2010). Lipopolysaccharides (LPS), a vital component of the outer membrane of Gram-negative bacteria, can stimulate the toll-like receptor 4 (TLR4)-mediated inflammatory signalling pathway (Yang et al. 2007; Guo et al. 2016; He et al. 2016). In addition, as a receptor of LPS, TLR4 is considered a fairly strong inflammatory stimulator (Bai et al. 2013). Among various signalling pathways concerning inflammatory reactions (Yang et al. 2007; Guo et al. 2016; He et al. 2016), the nuclear factor-κB (NF-κB) is a conserved transcription factor that plays a critical role in regulating the immune response to an infection (Karin and Delhase 2000; Huang et al. 2014). The NF-κB signalling pathway can be activated through TLR4 (Li et al. 2019). The NF-κB/Rel family in mammals includes c-Rel, Rel-B, p50, p52 and p65 (RelA). The best studied family member, P65, which is activated in response to various stimuli, is related to diverse cellular components, including inflammatory cytokines, chemokines and mediators of apoptosis (Yde et al. 2011; Basak et al. 2012). Additionally, activated P65 has been confirmed to be involved in multifarious inflammatory disorders, such as RIF (Barkett and Gilmore 1999; Fujihara et al. 2007). As cell proliferation, apoptosis and ECM deposition constitute major inflammatory responses, NF-κB is regarded as a key gene mediator in controlling this inflammation (Barkett and Gilmore 1999).

The phosphotidylinositol-3-kinase/Protein Kinase B (PI3K/AKT) pathway has been reported to play a crucial role in the fibrosis process through the modulation of various upstream and downstream factors, and by affecting fibroblast differentiation (Hsu et al. 2017; Du et al. 2019).

**Figure 1.**
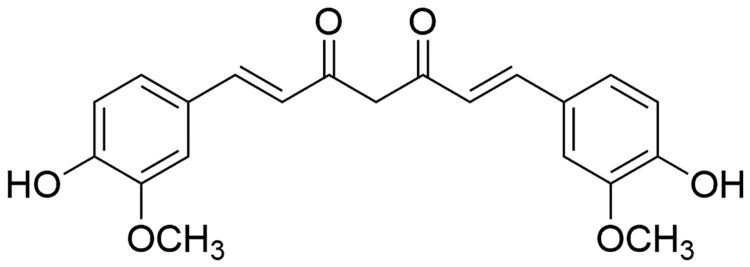
Chemical structure of curcumin.

Curcumin [(E,6E)-1,7-Bis(4-hydroxy-3-methoxyphenyl)hepta-1,6-diene-3,5-dione] (Figure 1), a chemical produced by the rhizomes of turmeric, has well-known antioxidative, antifibrogenic, anti-inflammatory and antiproliferative activities (Aggarwal and Sung 2009). Multiple studies have reported that curcumin has a latent therapeutic effect in multifarious pathological conditions, including hepatic, pulmonary and renal fibrosis (Kuwabara et al. 2006; Hamdy et al. 2012; Zhang et al. 2014). Soetikno et al. (2013) reported that curcumin attenuated oxidative stress, inflammation and RIF via the Nrf2-keap1 pathway in Sprague‐Dawley rats subjected to 5/6 nephrectomy. A study showed that curcumin facilitated proliferation of human renal proximal tubular (HK-2) cells, and antagonized TGF-β1-induced EMT via inhibition of the Akt/mTOR pathway (Zhu et al. 2017). Li et al. (2013) found that curcumin alleviated TGF-β1-induced EMT in HK-2 cells through ERK-dependent and then PPARγ-dependent pathways. Several studies have demonstrated that the TLR4/NF-кB and PI3K/AKT signalling pathways commonly regulate inflammation. For example, Piao et al. (2020) reported that picroside II promoted the intestinal barrier injury induced by severe acute pancreatitis in the rat model via inhibiting the TLR4-dependent PI3K/AKT/NF-κB signalling pathway. Zhao et al. (2014) demonstrated that the TLR4-mediated PTEN/PI3K/AKT/NF-κB signalling pathway was associated with the activation of a neuroinflammatory response in rat hippocampal neurons. As mentioned previously, increasing evidence has demonstrated that inflammation plays a vital role in the initiation and progression of RIF (Yiu et al. 2014; Lv et al. 2018). Nevertheless, to the best of our knowledge, no attempt has been made to correlate the role of curcumin in the TLR4/NF-кB and the PI3K/AKT signalling pathways. This study sought to shed light on this issue.

Herein, we explored the effects of curcumin on RIF and attempted to investigate the potential underlying mechanism. We examined the effect of curcumin on RIF *in vivo* and *in vitro*, via a unilateral ureteral obstruction (UUO) model as well as its effect on the transforming growth factor beta 1 (TGF-β1)-stimulated and the LPS-stimulated inflammatory responses in the human renal proximal tubular cells.

## Materials and methods

### Drug

Curcumin (98%) were purchased from Aladdin Industrial Corporation (Shanghai, China).

### Animals

Eighteen 18-24 g male C57 mice (8-weeks-old) were obtained from the Central Southern University. The UUO model was introduced as follows: the left flank skin was excised to reveal the left kidney and the ureter, which were ligated, before the wound layers were closed. Mice in the control group underwent the same surgical manipulation, except the left ureter was just exposed and not ligated. The eighteen mice were randomly assigned to the sham operation group, the UUO group, or the UUO + curcumin group, with six mice in each group. Mice in the UUO + curcumin group were administered curcumin by gastric gavage at a daily dose of 50 mg/kg from the first day (day of surgery) to the 14th day; all mice were sacrificed on the 14th day and both kidneys were collected. This study was permitted by the Ethics Committee Institute of Xiangya Hospital (No. 201703526), Central South University (Changsha, China).

### Cell culture

HK-2 cells were purchased from the American Type Culture Collection and maintained in Dulbecco’s modified Eagle’s medium (Thermo Fisher) containing 10% Foetal Calf Serum (Cyagen Bioscience, China) plus 1% penicillin and 1% streptomycin in an incubator (37 °C, 5% CO_2_). Cells were stimulated with either 10 ng/mL TGF-β1 with or without 10 ng/mL curcumin, or with 100 ng/mL LPS with or without 10 ng/mL curcumin, for 48 h. All cells were harvested for analyses.

### qPCR

Total RNA was isolated with TRIzol (Invitrogen). One microgram of the total RNA was utilized to generate the cDNA by reverse transcription with a RevertAid kit (Fermentas) according to the standard procedures. qPCR analyses were performed using the SYBR-Green qPCR SuperMix kit (ELK Biotechnology, China). The method of comparative threshold cycle (CT) was performed to quantify the relative gene expression. The formula 2^−ΔΔCT^ was used to estimate the fold change of the experimental sample relative to the control group. β-actin was utilized as an internal control. The relevant primer sequences have been listed in Table 1.

**Table 1.** The primer sequences for real-time PCR.

Gene	Sequences (5′–3′)
m-E-cadherin-Forward	CCTGTCTTCAACCCAAGCAC
m-E-cadherin-Reverse	CAACAACGAACTGCTGGTCA
m-β-actin-Forward	TCTTTGCAGCTCCTTCGTTG
m-β-actin-Reverse	TCCTTCTGACCCATTCCCAC
m-vimentin-Forward	TCTGTGTCCTCGTCCTCCTA
m-vimentin-Reverse	CGAGAAGTCCACCGAGTCTT
m-TLR4-Forward	TCTGGGGAGGCACATCTTCT
m-TLR4-Reverse	AGGTCCAAGTTGCCGTTTCT
m-TGF-β1-Forward	CAACAATTCCTGGCGTTACCTTGG
m-TGF-β1-Reverse	GAAAGCCCTGTATTCCGTCTCCTT

### Western blotting

Total cell protein was extracted with a radioimmunoprecipitation lysis buffer (Abcam). Protein (20 μg) was separated using 10% sodium dodecyl sulfate-polyacrylamide gel electrophoresis and electro-transferred onto polyvinylidene difluoride membranes (Sigma-Aldrich, Germany). The membranes were incubated in fat-free milk for 1 h, then incubated with primary antibody against NF-κB P65 (1:1000; Abcam, Cambridge, MA), IκBα(1:1000; Abcam), p-IκBα(1:1000; Abcam), p-P65(1:1000; Abcam), TGF-β1(1:500; Abcam), E-cad (1:1500; Abcam), ɑ-SMA (1:500; Abcam), vimentin (1:200; Abcam), TLR4 (1:100; Abcam), p-AKT (1:1000; Abcam), AKT (1:1000; Abcam), p-PI3K (1:1000; Abcam), PI3K (1:1000; Abcam), Histone H3(1:2000; Abcam), or β-actin (1:200; Abcam) at 4 °C overnight. Then, the membranes were incubated for 1 h with horseradish-peroxidase (HRP)-conjugated secondary antibody. A chemiluminescence reagent kit (Advanstar) was used to visualize the protein.

### Immunohistochemistry and histological examination

The kidney samples were excised, paraffin embedded and cut to 5 µm thick slices for histopathological examination and immunohistochemistry. The procedure for haematoxylin and eosin (H&E) and the Masson's trichrome staining has been described previously (Wang et al. 2020). Briefly, for Masson’s trichrome staining, the sections were deparaffinized, rehydrated, then stained with a haematoxylin iron solution (3 min), Ponceau–acid fuchsin solution (4 min), phosphomolybdic acid (1 min) and aniline solution (3 min). For H&E staining, sections were stained with haematoxylin (4 min) followed by differentiation using acid alcohol and counterstaining with eosin (2 min). For immunohistochemistry, the paraffin-embedded sections were dewaxed and dehydrated, and incubated in 3% hydrogen peroxide for 5 min, and a blocking buffer containing 5% bovine serum albumin for 30 min. Sections were incubated at 4 °C overnight with primary antibodies against NF-κB (1:200; Abcam), TGF-β1 (1:100; Abcam), E-cadherin (1:200; Abcam), vimentin (1:200; Abcam), TLR4 (1:50; Abcam) and α-SMA (1:1000; Abcam), before incubation with the HRP-conjugated secondary antibody. Images were acquired using a fluorescence microscope (Nikon, Japan).

### Cell viability assays

Cells (1 × 10^4^ cells/well) were plated in 96-well plates and treated with curcumin with or without TGF-β1 for 48 h. Cell viability was detected with a Cell Counting Kit 8 (CCK-8, MedChem Express, China).

### Enzyme-linked immunosorbent assay (ELISA)

An ELISA kit was used to detect the concentration of IL-6, IL-1β and TNF-α, according to the manufacturer’s instructions (Thermo Fisher).

### Renal function analysis

On the 14th day, all mice were sacrificed, and serum was obtained by centrifuging the blood collected from the ophthalmic artery. The serum levels of blood urea nitrogen (BUN) and serum creatinine (Scr) were determined immediately by colorimetric assays (Beyotime Biotechnology).

### Statistical analysis

All experiments were independently repeated three times. Data have been expressed as mean ± standard deviation. The Student’s *t*-test or one-way ANOVA were used to assess statistical significance. All analyses were computed with the SPSS Statistical software version 22.0. *p* < 0.05 was deemed statistically significant.

## Results

### Curcumin attenuated renal function and inflammation in UUO mice

**Figure 2.**
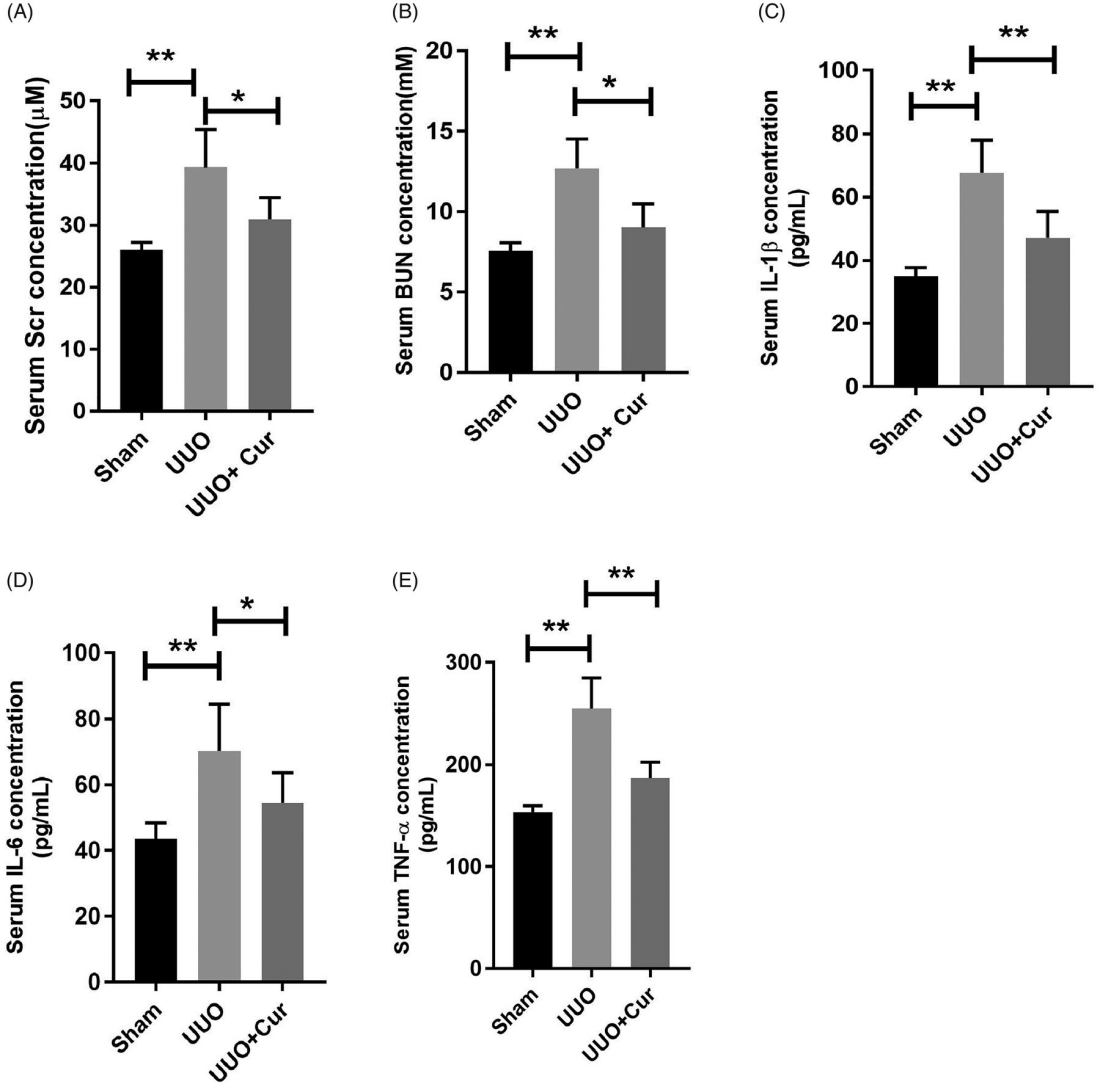
Curcumin attenuated renal function and inflammatory response in UUO mice. (A–B) Curcumin dramatically reduced the levels of blood urea nitrogen (BUN) and serum creatinine (Scr) in UUO mice (*n* = 6). (C–E) Curcumin notably decreased the serum levels of IL-6, IL-1β and TNF-α in UUO mice (*n* = 6). Cur, curcumin. *p < 0.05, **p < 0.01.

Given that Scr and BUN are two established parameters of renal function, we measured the serum levels of Scr and BUN in each group. As illustrated in Figure 2(A,B), the serum levels of Scr and BUN in the UUO group were notably enhanced compared with those in the sham group (39.28 ± 6.12 vs. 25.98 ± 1.23, *p* < 0.01; 12.68 ± 1.83 vs. 7.56 ± 0.51, *p* < 0.01). In contrast, the serum levels of Scr and BUN were significantly decreased in the curcumin treated group compared with those in the UUO group (30.91 ± 3.54 vs. 39.28 ± 6.12, *p* < 0.05; 9.04 ± 1.45 vs. 12.68 ± 1.83, *p* < 0.05).

To investigate the inflammatory changes in each group, serum levels of IL-1β, IL-6 and TNF-α were assessed using ELISA. As shown in Figure 2(C–E), the levels of the inflammatory factors were significantly increased in the UUO group compared to those in the sham group (67.63 ± 10.31 vs. 34.97 ± 2.71, *p* < 0.01; 70.28 ± 14.21 vs. 43.58 ± 4.81, *p* < 0.01; 254.71 ± 30.21 vs. 153.4 ± 6.34, *p* < 0.01; IL-1β, IL-6 and TNF-α, respectively). In contrast, treatment with curcumin dramatically reduced the levels of IL-1β, IL-6 and TNF-α compared to those in the UUO group (47.13 ± 8.31 vs. 67.63 ± 10.31, *p* < 0.01; 54.49 ± 9.19 vs. 70.28 ± 14.21, *p* < 0.05; 186.72 ± 15.81 vs. 254.71 ± 30.21, *p* < 0.01; respectively). These data suggested that curcumin treatment improved renal function and alleviated the inflammatory response induced by UUO.

### Curcumin suppressed ECM deposition in UUO mice

**Figure 3.**
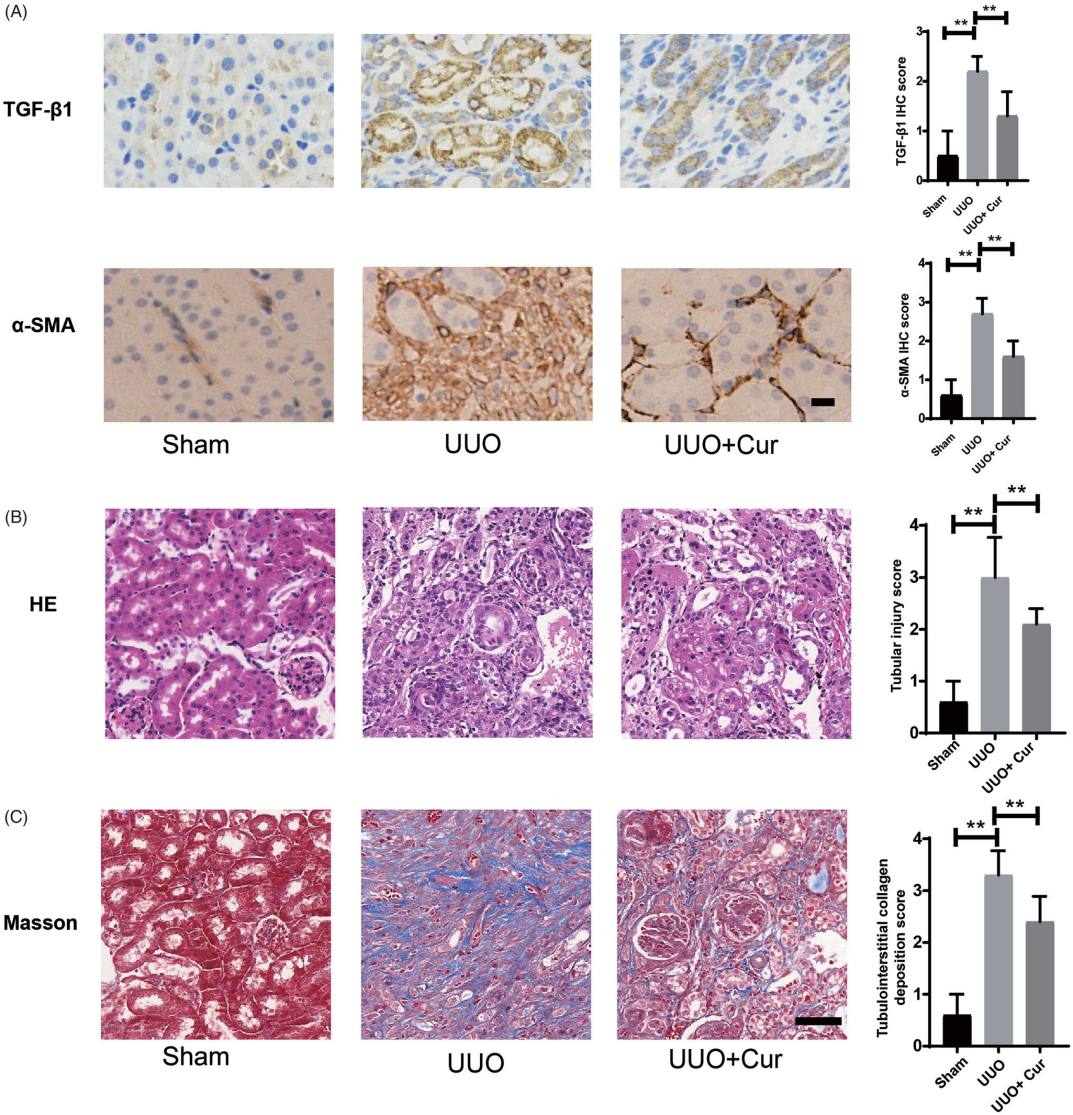
Curcumin suppressed ECM deposition in UUO mice. (A) Immunochemical staining of TGF-β1 and α-SMA in mouse kidney tissue (200× magnification, bar = 50 μm). The IHC evaluation was performed by two independent observers, who were blind to the slice. The staining intensity was scored as (1) 0 point: negative; (2) 1 point: weak positive; (3) 2 points: moderated positive; (4) 3 points: strong positive. (B) Representative H&E staining images (400× magnification, bar = 50 μm). Ten tubulointerstitial non-overlapping fields were randomly chosen. The score was based on the percentage of damaged tubules in the renal cortex: (1) 0 point: normal; (2) 1 point: less than 10% of the renal cortex; (3) 2 points: 10–25% of the renal cortex; (4) 3 points: 25–50% of the renal cortex; (5) 4 points: 50–75% of the renal cortex; (6) 5 points: more than 75% of the renal cortex (Leemans et al. 2005). (C) Representative Masson’s trichrome staining images (400× magnification, bar = 50 μm). Ten tubulointerstitial non-overlapping fields were randomly chosen. The score was based on the percentage of positively stained cells: (1) 0 point: no staining; (2) 1 point: less than 25% staining; (3) 2 points: 25–50% staining; (4) 3 points: 50–75% staining; (5) 4 points: more than 75% staining (Lin et al. 2005). Cur, curcumin. *p < 0.05, **p < 0.01.

To evaluate the effects of curcumin on the ECM deposition in UUO mice, histopathological and immunohistochemical analyses were performed. As illustrated in Figure 3(A), the expression levels of TGF-β1 and α-SMA in the UUO group were dramatically enhanced compared to that in the Sham group. However, treatment with curcumin significantly decreased their expression compared to that in the UUO group. H&E staining revealed that the interstitial space was widened, inflammatory cell infiltration and peripheral exudation were increased, glomerular capillaries were dilated and renal tubular cells were atrophied in the UUO group; the morphological changes induced by UUO significantly improved with curcumin treatment (Figure 3(B)). Deposition of ECM collagen was significantly enhanced in the UUO group compared to that in the sham group, whereas the introduction of curcumin reduced this increased deposition induced by UUO, as evidenced by Masson’s trichrome staining (Figure 3(C)). Besides, the tubular injury score and tubulointerstitial collagen deposition score were introduced to analyze H&E staining and Masson’s trichrome staining images as a semi-quantitative analysis, respectively. As illustrated in Figure 3(B,C), the tubular injury score and tubulointerstitial collagen deposition score were significantly increased in the UUO group compared to those in the sham group. In the curcumin-treated group, the two increased scores induced by UUO were remarkably reduced compared to those in the UUO group. Accordingly, these results revealed that curcumin inhibited ECM deposition induced by UUO.

### Curcumin restrained EMT and suppressed inflammation via the TLR4/NF-κB pathway in UUO mice

**Figure 4.**
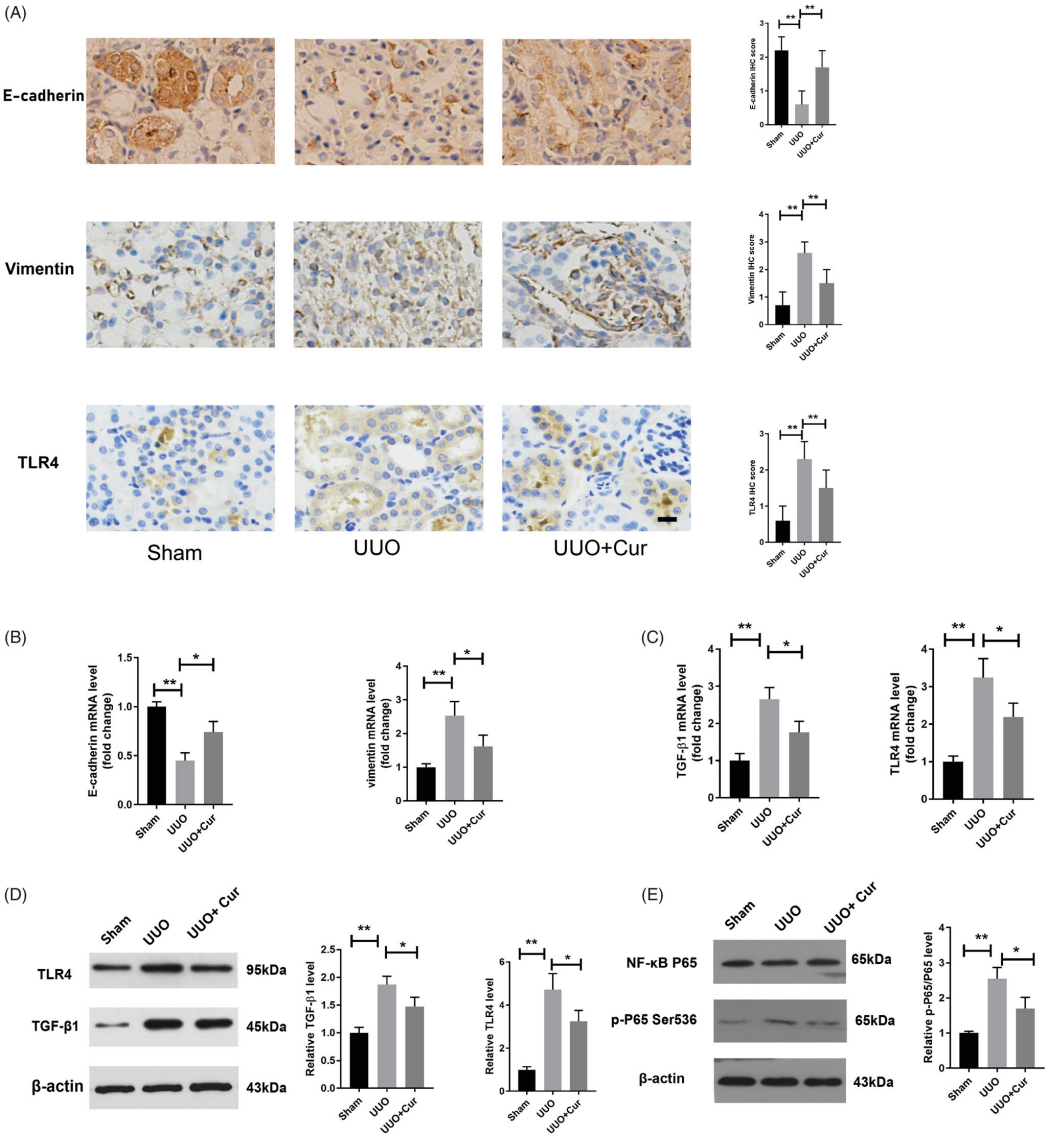
Curcumin suppressed EMT and inflammation via the TLR4/NF-κB pathway in UUO mice. (A) Expression of E-cadherin, vimentin and TLR4 in mouse renal sections, detected by immunochemical staining. (200× magnification, bar = 50 μm). The IHC evaluation was performed by two independent observers, who were blind to the slice. The staining intensity was scored as (1) 0 point: negative; (2) 1 point: weak positive; (3) 2 points: moderated positive; (4) 3 points: strong positive. (B) The expression of E-cadherin and vimentin in UUO mice, as evidenced by qPCR (*n* = 4). (C–D) qPCR and western blotting analyses of the expression of TGF-β1 and TLR4 in UUO mice (*n* = 4). (E) The expression of NF-κB P65 in the renal tissue, detected by western blotting (*n* = 4). Cur, curcumin. *p < 0.05, **p < 0.01.

As illustrated in Figure 4(A,B), curcumin suppressed EMT via notably reducing the increased expression of vimentin induced by UUO, along with promoting the reduced expression of E-cadherin also induced by UUO, as evidenced by immunohistochemistry and qPCR. The inflammatory response is reportedly involved in the progression of RIF, along with TLR4, which promotes the increased expression of inflammatory cytokines (Li et al. 2019). To explore the anti-inflammatory effects of curcumin on RIF, immunohistochemistry, qPCR and western blotting were performed to measure the expression levels of TLR4 and NF-κB. As illustrated in Figure 4(A), the expression of TLR4 dramatically increased in the UUO group compared to that in the Sham group, while treatment with curcumin significantly decreased the expression of TLR4 compared to that in the UUO group. Furthermore, in the curcumin-treated group, the expression levels of TGF-β1, TLR4 and p-P65 were dramatically decreased compared to those in the UUO group, as evidenced by qPCR and western blotting (Figure 4(C–E).

### Curcumin repressed the TGF-β1 induced EMT in HK-2 cells via the TLR4/NF-κB and the PI3K/AKT pathways

**Figure 5.**
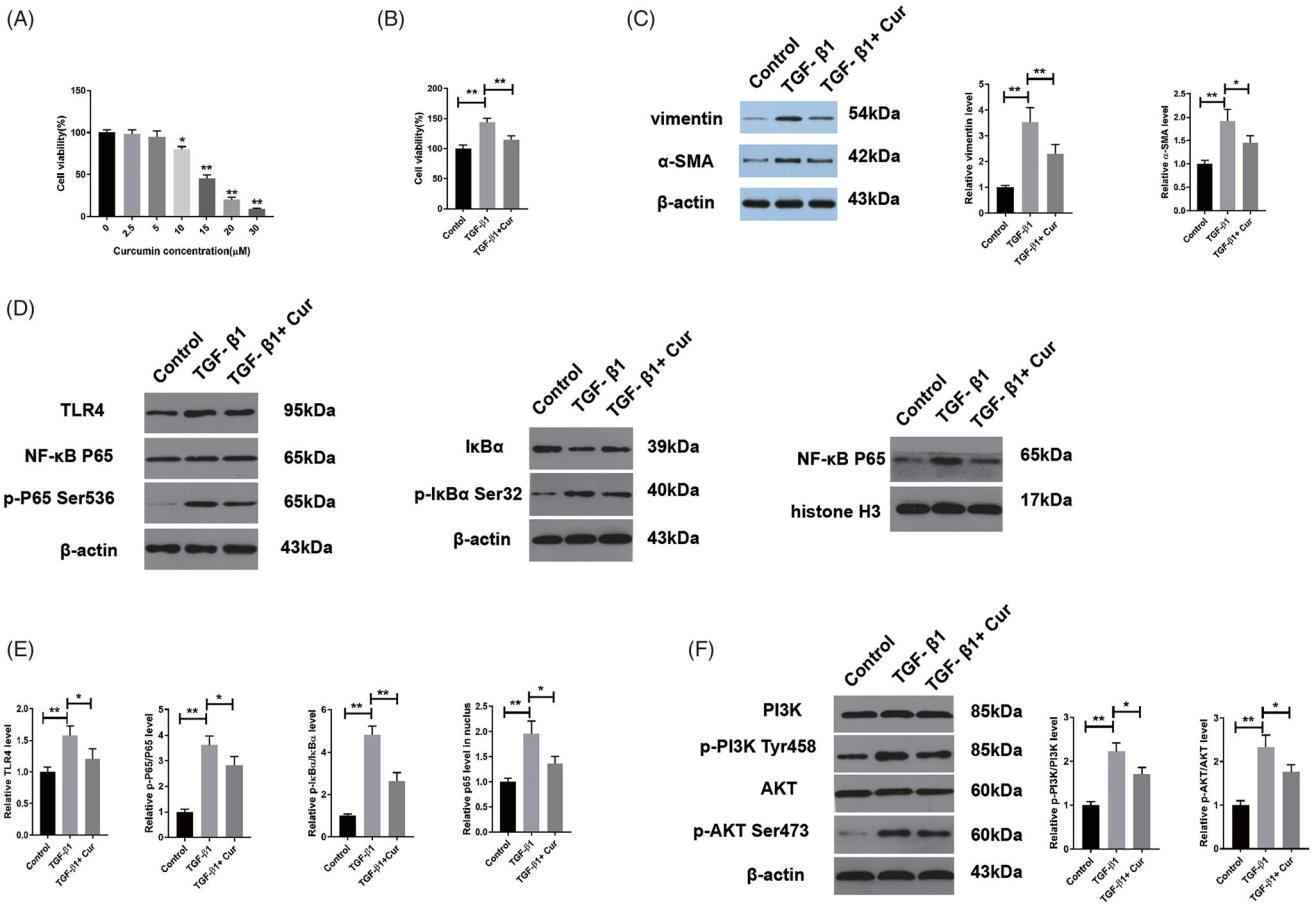
Curcumin repressed the TGF-β1-induced EMT in HK-2 cells. (A) Viability of HK-2 cells treated with various concentrations of curcumin, as detected using CCK-8 (*n* = 3). (B) CCK-8 analysis of HK-2 cell viability (*n* = 3). (C) Western blot analyses of curcumin and TGF-β1 in HK-2 cells (*n* = 3). (D–F) Curcumin suppressed the TGF-β1-induced increase in TLR4, p-P65, p-IκBα, p-PI3K and p-AKT expression in HK-2 cells, as evidenced by western blotting (*n* = 3–5). Cur, curcumin. *p < 0.05, **p < 0.01.

To investigate the effect of curcumin on RIF *in vitro*, HK-2 cells were treated with various concentrations of curcumin. Curcumin concentrations of >10 μM caused a sharp decrease in cell viability; thus, 10 μM curcumin was chosen for subsequent experiments (Figure 5(A)). TGF-β1 is an important profibrotic mediator of EMT; according to numerous studies, 10 ng/mL TGF-β1 is sufficient to induce an EMT in HK-2 cells over a 48 h period (Wang et al. 2014; You et al. 2016; Xu et al. 2019). Curcumin significantly reduced the increased cell viability caused by TGF-β1, as demonstrated by the CCK-8 assay (Figure 5(B)). Next, western blotting was utilized to explore the mechanism behind curcumin suppressing EMT in TGF-β1-induced HK-2 cells. Treatment with curcumin inhibited TGF-β1-induced EMT via suppression of vimentin and α-SMA (Figure 5(C)). As illustrated in Figure 5(D,E), TGF-β1 facilitated the phosphorylation and translocation of p65 from the cytoplasm to nucleus, accompanied with phosphorylation and degradation of IκBα in the cytoplasm, which were all antagonized by the treatment with curcumin. Besides, the expression of TLR4 was significantly increased in the TGF-β1 group compared to that in the control group, while the administration of curcumin reversed this increase induced by TGF-β1 (Figure 5(D,E)). Furthermore, to evaluate the potential mechanism, the protein expression levels of the PI3K/AKT pathway were measured. As shown in Figure 5(F), p-PI3K and p-AKT levels were dramatically elevated in TGF-β1-induced cells compared to those in the control cells. However, the TGF-β1-induced increases in PI3K and AKT phosphorylation were suppressed by treatment with curcumin.

### Curcumin inhibited the LPS-stimulated inflammatory response by suppressing the TLR4/NF-κB and the PI3K/AKT pathways

**Figure 6.**
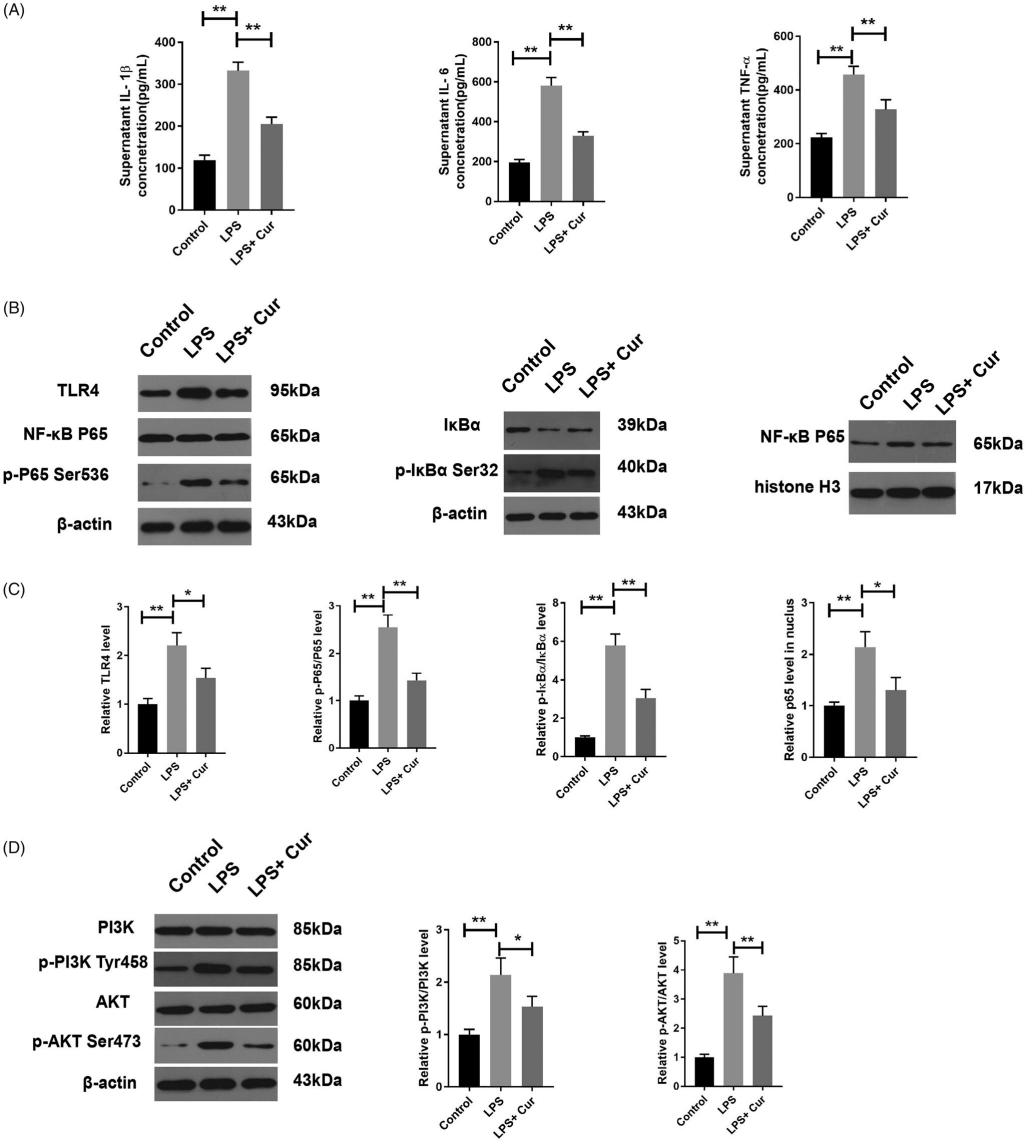
Curcumin inhibited the LPS-stimulated inflammatory response. (A) ELISA analyses of the concentration of IL-1β, IL-6 and TNF-α in the supernatant of HK-2 cell culture medium (*n* = 3). (B–D) Curcumin suppressed the LPS-induced increase in TLR4, p-P65, p-IκBα, p-PI3K and p-AKT in HK-2 cells, as evidenced by western blotting (*n* = 3–4). Cur, curcumin. *p < 0.05, **p < 0.01.

LPS was introduced into the HK-2 cells to confirm that the anti-inflammatory effect of curcumin was exerted via the TLR4/NF- κB and PI3K/AKT pathways. To explore the changes in inflammation in the LPS-stimulated HK-2 cells, the levels of IL-1β, IL-6 and TNF-α were detected by ELISA. As demonstrated in Figure 6(A), these inflammatory cytokines were dramatically increased in the LPS group compared to those in the control cells (332.5 ± 20.2 vs. 119.2 ± 11.7, *p* < 0.01; 581.9 ± 40.2 vs. 194.8 ± 15.7, *p* < 0.01; 457.6 ± 30.2 vs. 223.5 ± 14.3, *p* < 0.01; IL-1β, IL-6 and TNF-α, respectively), while treatment with curcumin significantly decreased the levels of IL-1β, IL-6 and TNF-α compared to those observed upon LPS treatment (205.7 ± 16.1 vs. 332.5 ± 20.2, *p* < 0.01; 329.3 ± 20.1 vs. 581.9 ± 40.2, *p* < 0.01; 327.9 ± 36.1 vs. 457.6 ± 30.2, *p* < 0.01, respectively). Additionally, compared with the control cells, the phosphorylation and translocation of p65 from the cytoplasm to nucleus, as well as the phosphorylation and degradation of IκBα in the cytoplasm, were dramatically increased by LPS induction. These activities were reversed by curcumin treatment (Figure 6(B,C)). Furthermore, the expression of TLR4 was remarkably enhanced in the LPS-induced HK-2 cells compared to that in the control cells, while treatment with curcumin reversed this increase induced by LPS (Figure 6(B,C)). Furthermore, LPS dramatically enhanced the phosphorylation of PI3K and AKT proteins compared with that in control cells. However, treatment with curcumin significantly decreased the phosphorylation of PI3K and AKT proteins compared with that observed in LPS-induced HK-2 cells (Figure 6(D)).

## Discussion

Increasing evidence has demonstrated that curcumin has antioxidative, antifibrogenic, anti-inflammatory and antiproliferative effects (Aggarwal and Sung 2009; Soetikno et al. 2013; Zhou et al. 2014). Zhou et al. (2014) found that curcumin ameliorated RIF by lessening fibroblast proliferation and ECM deposition mediated by the PPAR-g and the Smad-dependent TGF-β1 pathway. Soetikno et al. (2013) demonstrated that curcumin could effectively ameliorate oxidative stress, inflammation, as well as RIF through regulation of the Nrf2-Keap1 pathway. Herein, curcumin was shown to prevent an ECM deposition, an EMT and renal inflammation processes, as well as reverse the poor renal function in a UUO model. Additionally, curcumin notably reversed the TGF-β1-induced EMT by suppressing the TLR4/NF-κB and the PI3K/AKT pathways in the TGF-β1-induced HK-2 cells. Additionally, LPS was administrated to obtain a renal inflammatory model. Curcumin was first found to inhibit the LPS-stimulated inflammatory response through repression of the TLR4/NF-κB and the PI3K/AKT pathways.

Activation of TGF-β1 is deemed a vital event in the progression of EMT, which ultimately leads to RIF (Border and Noble 1997; Zhang et al. 2019). TGF-β1 was introduced to stimulate the HK-2 cells to acquire a RIF model. Even though it is common knowledge that TGF-β1 induces EMT by activating the Smad pathway, TGF-β1 could activate other non-Smad-dependent signalling pathway as well, such as the Akt/mTOR pathway (Zhu et al. 2017), the ERK-dependent and PPARγ-dependent pathways (Li et al. 2013), and the PI3K/Akt/ERK signalling pathway (Zhang et al. 2019). In the present study, the administration of TGF-β1 dramatically promoted the cell viability and EMT, curcumin treatment reversed these affects via the TLR4/NF-κB and the PI3K/AKT pathways, illustrating the mechanism behind curcumin’s anti-EMT activity.

It has been shown that inflammation is closely linked to fibrosis and the inflammatory response promotes RIF (Holdsworth and Summers 2008; Li et al. 2019). TLR4 is strongly associated with inflammation (Bai et al. 2013), and it has been shown that TLR4-deficient UUO mice have less renal fibrosis and a reduced expression of the inflammatory cytokines compared to the normal UUO mice (Pulskens et al. 2010). In progression, many factors have been reported as fibrogenic factors in the kidneys. The conserved transcription factor, NF-κB, promptly responds to proinflammatory stimuli and plays a vital role in inflammatory and immune responses (Karin and Delhase 2000). Additionally, NF-κB activation dramatically promotes RIF and fibroblast activation (Li et al. 2011). Moreover, evidence has confirmed that NF-κB is a crucial central mediator in EMT (Miyajima et al. 2003; Chua et al. 2007; Huang et al. 2013).

The TLR4/NF-κB signalling pathway has been found to be involved in several fibrosis processes (Liu et al. 2019; Jin et al. 2020). Liu et al. (2019) found that scoparone ameliorated inflammation and fibrosis in non-alcoholic steatohepatitis via inhibition of the TLR4/NF-κB pathway in mice. Jin et al. (2020) found that crocin alleviated myocardial fibrosis induced by isoprenaline by repressing inflammatory cytokine expression and apoptosis via the TLR4/NF-κB signalling pathway. In our study, curcumin alleviated EMT and the inflammatory response in the TGF-β1-induced HK-2 cells and the LPS-induced HK-2 cells, respectively, via the TLR4/NF-κB pathway. Li et al. (2019) performed a study to explore the potential mechanism of salidroside (Sal) in anti-inflammation and renal protective effect. They found the treatment of Sal improved kidney function, alleviated the ECM deposition and relieved the protein expression levels of EMT markers in UUO mice and TGF-β1-induced HK-2 cells. Additionally, Sal treatment dramatically reduced the release of inflammatory cytokines (IL-1β, IL-6 and TNF-α) and inhibited the TLR4/NF-κB and MAPK signalling pathways in LPS-induced HK-2 cells. These results are consistent with our study. Firstly, we both used TGF-β1 and LPS to acquire a renal fibrosis model and inflammatory model for further mechanism measurement, respectively. Secondly, TLR4/NF-κB were both confirmed to play a critical role in the treatment (Sal or curcumin) alleviating EMT and the inflammatory response.

Liu et al. (2018) carried out a study to explore the effect of human umbilical cord-derived mesenchymal stem cell (hucMSC) conditioned medium (CM) on renal tubulointerstitial inflammation and RIF. Their results revealed that hucMSC-CM had protective effects against RIF induced by UUO and that hucMSC-CM achieved its effects of anti-inflammation via suppression of the TLR4/NF-κB signalling pathway. The TLR4/NF-κB signalling pathway was demonstrated to be involved in the mechanism of curcumin/hucMSC-CM anti-inflammation and anti-EMT in both that study and our study. However, they only utilized TGF-β1 to induce the HK-2 cells to evaluate the effect of hucMSC-CM on inflammatory responses, nevertheless, both TGF-β1-induced HK-2 cells and LPS-induced HK-2 cells were used in our study. Given that LPS is a ligand of TLR4 (Bai et al. 2013), it is more feasible to use TGF-β1 and LPS to induce cells for further measurement of inflammatory cytokines.

The PI3K/AKT signalling pathway plays an eventual role in cell differentiation, proliferation, survival, apoptosis, angiogenesis, migration and EMT (Ye et al. 2012; Zhu et al. 2018; Liang et al. 2019). Our study showed that activation of the PI3K/AKT pathway in HK-2 cells induced by TGF-β1 and LPS was dramatically reduced by the administration of curcumin, suggesting that the curcumin-induced inhibitory effect on EMT, ECM deposition and inflammation, may target the PI3K/AKT pathway. Zhu et al. (2017) performed a study to investigate the mechanism of curcumin on TGF-β1-induced EMT *in vitro*. Their data suggested that curcumin facilitated HKC proliferation and antagonized TGF-β1-induced EMT via inhibition of the activity of the Akt/mTOR pathway. AKT was confirmed to play a critical role in their study and our study. However, they only performed an *in vitro* experiment, there is no doubt it would be more feasible to preform both *in vivo* and *in vitro* experiments. EMT and angiogenesis function are two pivotal events in the development of cancer (Su et al. 2015). Jiao et al. (2016) performed a study using hepatocyte growth factor (HGF) to stimulate lung cancer cells. The results suggested that curcumin effectively inhibited HGF-induced EMT and angiogenesis via targeting c-Met and suppressing the PI3K/Akt/mTOR pathways. Therefore, PI3K/Akt was confirmed to play a critical role in the alleviation of EMT by curcumin. Nevertheless, a PI3 kinase inhibitor LY294002 was used in their study to precisely research the role of the pathway in the process, which would make their results more precise.

## Conclusion

We reported that curcumin effectively prevented EMT, ECM deposition and inflammation via the TLR4/NF-κB and PI3K/AKT signalling pathways. Consequently, we propose that curcumin may serve as a potential therapeutic agent in RIF treatment.
